# A dataset for classifying phrases and sentences into statements, questions, or exclamations based on sound pitch

**DOI:** 10.1016/j.dib.2025.111826

**Published:** 2025-06-24

**Authors:** Ayub Othman Abdulrahman, Shanga Ismail Othman, Gazo Badran Yasin, Meer Salam Ali

**Affiliations:** Department of Computer Science, College of Science, University of Halabja, Kurdistan Region, F.R., Halabja, Iraq

**Keywords:** Statement, Questions, Exclamations, Sound classification, Pitch of sound, And natural language processing (NLP)

## Abstract

Speech is the most fundamental and sophisticated channel of human communication, and breakthroughs in Natural Language Processing (NLP) have substantially raised the quality of human-computer interaction. In particular, new wave of deep learning methods have significantly advanced human speech recognition by obtaining fine-grained acoustic cues including pitch, an acoustic feature that can be a critical ingredient in understanding communicative intent. Pitch variation is in particular important for prosodic classification tasks (i.e., statements, questions, and exclamations), which is crucial in tonal and low resource languages such as Kurdish, where intonation holds significant semantic information. This paper presents the dataset of the Statements, Questions, or Exclamations Based on Sound Pitch (SQEBSP) which contains 12,660 professionally-recorded speech audio clips by 431 native Kurdish speakers who reside in the Kurdistan Region of Iraq.

Regarding utterances, 10 new phrases were articulated by each speaker per three prosodic categories: statements, questions, and exclamations. All utterances were digitized at 16 kHz and then manually checked for correctness concerning pitch-based classification. The dataset contains equal representation from all three classes, about 4200 samples per class, and metadata such as speaker gender, age group, and sentence identifiers.

The original audio files, alongside resources like Mel-Frequency Cepstral Coefficients (MFCCs) and waveform visualizations, can be found on Mendeley Data. The dataset offered has significant advantages for formulating and testing pitch-based speech classification algorithms, furthers the work on pronunciation modelling for languages lacking sufficient resources. It furthermore, aids in developing speech technologies sensitive to dialects.

Specifications Table

**Table:** 

Subject	Computer Sciences
Specific subject area	The dataset provided emphasises learning and understanding sound phrases and sentences. It helps linguists and academics identify pitch changes in a particular language; therefore, it serves as a useful tool. Furthermore, it significantly improves voice recognition and natural language processing (NLP).
Type of data	Sound (wave), Matrix (MFCC)
Data collection	The “Classifying Phrases and Sentences into Statements, Questions, or Exclamations” dataset records three primary categories of sentences: Statements, Questions, and Exclamations. Samples were recorded from 431 participants using a highly sensitive microphone to guarantee clarity. Each audio sample is just one second long and has been converted to wave format. Every sample has also been converted to a matrix to facilitate further research.
Data source location	Researchers from the University of Halabja, IKR/IRAQ, recorded and collected the samples; all participants were Kurdish from Iraqi Kurdistan Region.
Data accessibility	Repository name: Data Mendeley Data identification number: 10.17632/267dh85vpw.1 Direct URL to data: https://data.mendeley.com/datasets/267dh85vpw/1 Instructions for accessing these data: Abdulrahman, Ayub; Othman, Shanga; Yasin, Gazo; Ali, Meer (2025), “A Dataset for Categorizing Phrases and Sentences as Statements, Questions, or Exclamations Depending on Sound Pitch”, Mendeley Data, V1, doi:10.17632/267dh85vpw.1 [1].
Related research article	None

## 1 Value of the Data

• **The first Kurdish pitch-annotated corpus:** This dataset provides the only publicly accessible pitch-annotated speech dataset for Kurdish language including 12,660 one-second sounds exactly classified into statements, questions, and exclamations.
• **Quantitative Analysis of Intonation**
• Researchers may use the exact alignment of identical lingual data across three pitch situations to examine contour variations, create novel pitch-tracking algorithms, and enhance intonational phonology models.
• **Understanding How We Use Pitch in Speech:** The dataset gives researchers the possibility to study the influence of different pitch variants on our natural expressions of statements, questions, and exclamations. This dataset is highly significant in the research of speech patterns as it can be beneficial for the linguists who specialize in tone and sentence structure.
• **Helping Language and Dialect Studies:** This dataset makes it possible to be used to compare the articulated norms of pronunciation among the different languages and dialects which are utilized by different linguistic communities. It can moreover, help researchers investigate how the pitch changes in various linguistic contexts which this in turn, leads to better multilingual speech processing models.
• **Providing a Standard Benchmark for Future Research:** This dataset distinguishes itself as the most accurate data for researchers in the field of speech classification when they are in the process of developing new models. By adopting it as a benchmark, researchers will be able to compare their experimental methods and refine them, thereby advancing the field of speech technology.
• **Benchmark Performance:** To the best of researchers’ knowledge, only two prior studies have reported overall accuracy in classifying statements, questions, and exclamations based on pitch., with Cenceschi et al [2], showing 68% accuracy on Italian data using a biLSTM, and Effendy et al [3], achieving 89.1% on Indonesian data with an MFCC-based network. Our evaluation shows that a multi-modal fusion model trained on SQEBSP reaches an accuracy of 97.91%, which is much higher than the previous studies and demonstrates that this dataset is effective for creating high-performing prosody classifiers.
• **Practical Implementation**: Sample code (https://github.com/Ayubad/Statements-Questions-or-Exclamations-based-on-sound-pitch-SQEBSP-Kurdish-dataset/tree/main) was provided to demonstrate the process of loading WAV files, precomputed MFCC matrices, and spectrogram images directly from the raw data for comprehensive training and inference.
• **Application scenarios:**
  ○ **Voice Assistants:** Employ SQEBSP to train intent-detection modules that use pitch contours to differentiate user statements, questions, and exclamations in Kurdish language conversational agents.
  ○ **Telemedicine Alerts:** Create systems that activate voice-based notifications when patients' vocalisations (e.g., statements of discomfort) surpass specified pitch thresholds.
  ○ **Educational Tools:** Develop modules for pronunciation and prosody feedback in language-learning programs, using balanced classrooms for effective model training.

## 2 Background

Among humans, speech is an essential form of communication and the most natural and effective means of sharing knowledge. Researchers have always been attempting to understand and explore different ways to enhance the connection between people and computers [4]. Natural language processing (NLP) is a branch of artificial intelligence and language study invested in enabling computers to understand words or statements expressed in human languages. One of the most fascinating features of NLP is that it builds up to human language understanding. NLP is associated with many ideas and methods addressing the issue of natural language communication with the machines [5]. Machine learning techniques have facilitated the implementation of sound recognition systems. The development of machine learning methods (including deep learning approaches) has shown remarkable ability in extracting high-level features that have allowed algorithms to efficiently learn complicated level features from raw input data [6].

The pitch of sound affects methods of categorizing phrases and sentences in natural languages. Pitch's analysis and extraction methodologies enable speech and music recognition, sound segregation, and several other acoustic functions [7]. Moreover, speech signals demonstrated variability across people due to acoustic characteristics such as accent, vocal tract parameters, gender, and emotional expression, allowing scientists and scholars to distinguish among speakers [8].

Pitch of sound guides sentence and phrase categorisation algorithms to group spoken languages. Age, gender, and sentence type presence affect the speech recognition accuracy of the speaker [9]. Including a variation detection system, helps speech recognition systems to adapt variances in voice. Speeches are often classified into four types: questions, statements, exclamations, and demands. Information on speech types is crucial for understanding speaking [3]. In the Kurdish language, a singular collection of words or phrases can refer to different forms of communication, including statements, questions, or exclamations. The differentiating characteristic for identifying these types of content is not the text or words themselves, but rather the pitch and tone used throughout the way they are spoken. Therefore, the pitch of spoken sound plays a crucial role for expressing a specific message.

## 3 Data Description

The dataset categorises sound expressions of phrases and sentences in the Kurdish language as statements, questions, or exclamations based on sound pitch (SQEBSP). Pitch of sound is the lowness or highness of a sound that depends on the frequency of the sound waves. Pitch is very important in speech for communicating many meanings, emotions, or purposes. Recent study has shown pitch-based connections with several conceptual and behavioural traits [10]. Depending on the pitch variation when speaking, the same phrase could be understood as a statement, questions, or exclamation [11]. Unfortunately, to the best of the researchers’ knowledge, there is no available dataset for classifying sound expressions into statements, questions, or exclamations based on sound pitch in the Kurdish language. This deficiency extends to surrounding languages such as Arabic, Persian, Indian, and Turkish, as well as English and Spanish; the only two references identified are [2] and [3].

Every model developed using a machine learning method requires data collection first. A number of researchers at the Computer Science Department of the University of Halabja gathered the proposed dataset. Throughout the data-collecting process, the usual methods and guidelines were used. Additional conditioning elements included in the dataset were speaker age and gender. Fortunately, including these restrictions during data collection, could provide a more generalizable model. A total of 431 KRI/Iraq participants participated in the recordings. Table 1 shows the participants' statistics, particularly their gender and age.

**Table 1:** The demographic information of the participants, including their gender and age.

Participants	Under 18	18-25	26-32	33-40	Over 40	Total
Male	30	110	17	25	8	190
Female	17	177	22	15	10	241
Total	47	287	39	40	18	431

Each participant was given instructions to read the phrases shown in Table 2 as a statement, questions, and exclamation using varying tone of voice. Each participant had thirty sound samples captured overall from the recordings, which were produced with a top-notional microphone (Rode NT1).

**Table 2:** Phrases and sentences employed in this research.

Each category (Statements, Questions, or Exclamations) has 10 subfolders, with each subfolder representing a specific phrase or sentence that is illustrated in Table 2. Each subfolder has between 420 and 430 audio recordings and therefore, each class has about 4,200 audio samples, and the whole dataset contains a total of 12,660 sound recordings which is indicated in Table 3.

**Table 3:** Audio recording distribution by expression category and corresponding phrase.

Phrase No.	Statements	Questions	Exclamations	Total
1	422	423	416	1,261
2	425	423	421	1,269
3	424	424	422	1,270
4	426	425	420	1,271
5	422	424	420	1,266
6	423	423	420	1,266
7	421	423	418	1,262
8	423	419	420	1,262
9	424	423	420	1,267
10	423	421	422	1,266
Total	4,233	4,228	4,199	12,660

**Fig. 1:**
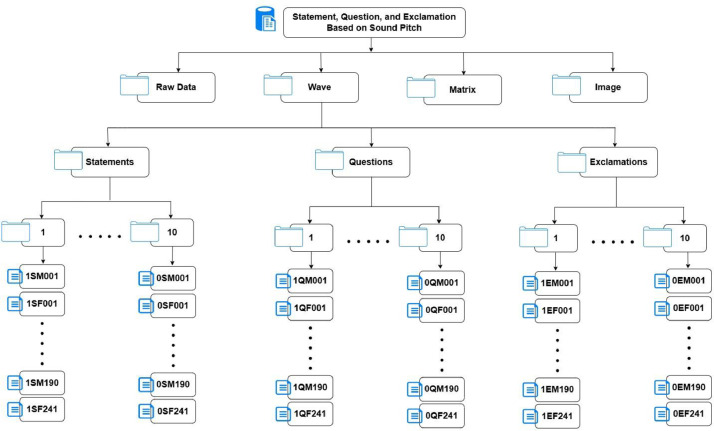
The proposed dataset architecture.

The dataset, titled “A Dataset for the Classification of Phrases and Sentences into Statements, Questions, or Exclamations Based on Sound Pitch,” is organized into four main subdirectories as shown in Fig. 1: Raw Data (original recordings), Wave (WAV files), Matrix (matrix files), and Image (visual representations of the waveform). Each of these primary directories is further divided into three subdirectories corresponding to the expression types: Statements, Questions, and Exclamations. Within each expression type, there are 10 folders representing the 10 different sentences or phrases, and each folder contains approximately 420 files associated with the Statements, Questions, and Exclamations.

All recordings were processed at a 16 kHz sampling rate with 16-bit PCM encoding and recorded in both OGG and WAV formats to guarantee repeatability and ease of use. Each file corresponds to the naming standards: sentence_id, expression_type, gender, speaker_id, and file extension. The filename, 4QF231.ogg, indicates Phrase 4, Question form, Female speaker 231 in raw OGG format. The dataset includes four primary folders: Raw (original OGG files), WAV (converted WAV recordings), Matrix (12-dimensional MFCC .txt files), and Image (1000 × 600 Image representation of wave signal .png files). Each folder is further separated into three expression classifications and ten sentence-ID subfolders, as seen in Fig. 1.

The data article includes original audio recordings in OGG format, MFCC feature matrices, WAV-format audio files, and graphic representations of the waveforms in PNG format. The dataset offers raw audio data, allowing researchers to extract particular features and transform the data into forms suitable for their analyses (such as .npy files for extracted features, MFCCs, spectrogram image in .png, or other representation types). All of this can be readily accomplished using the raw data available through the dataset link.

## 4 Experimental Design, Materials and Method

The present article indicates the methodology used for the collection, labelling, preprocessing, and structuring of the “Statements, Questions, or Exclamations Based on Sound Pitch” (SQEBSP) Kurdish dataset. The dataset is designed to facilitate future research on pitch-based speech identification, especially in low-resource languages.

The primary goal in the present work was to collect speech data to classify sentences or phrases as statements, questions, and exclamations. This paper shows improvements in the area of present-day natural language processing (NLP). The purpose of the study was to improve the understanding of the Kurdish language and to inspire technologies such as voice recognition, automated transcription, and virtual assistants able to interact with Kurdish speakers.

From 431 participants of all ages and genders, a team at the University of Halabja, IKR/Iraq, gathered the SQEBSP dataset. Different technologies were used to convert voice recordings into waveform representations. Each speaker's recordings have been split into one-second segments at a frequency range of 16 kHz, mono channel, and 16-bit resolution. Each category contains more than 4,200 sound elements, while the complete dataset consists of 12,660 sound files.

### 4.1 Data Collection

Data collection is a fundamental prerequisite for systems developed using machine learning algorithms. Machine learning is dependent on having accessibility of a dataset [12]. Researchers can more effectively evaluate and recognize different types of phrases in speech using today's techniques and instruments powered by machine learning and artificial intelligence. These advancements enhance the preservation of linguistic variety and intercultural communication by enhancing the understanding of sentence patterns.

Stringent standards were applied throughout the data-collecting process to guarantee data quality maintenance. The researchers conducted a thorough assessment using accurate recording techniques to identify and resolve any conflicts or anomalies in the recorded sound elements. A subset of the recorded sound samples passed an accurate assessment carried out by researchers with specific training in order to improve the reliability of the dataset. Those samples showing anomalies or poor quality, were found and subsequently either excluded from the dataset or reconfigured.

In addition to quiet lab-style recordings, some samples were gathered in open or semi-controlled environments, providing real background noise like traffic, wind, and distant talk. Other researchers may immediately access these noisy recordings via the Raw Data directory by sampling files inside the dataset. This range of sound settings improves the development and assessment of models robust to real-world noise.

The following phases describe the process of data generation:

➢ Participants recorded ten phrases in three prosodic modalities (statements, questions, and exclamations).
➢ Each speech was separated into 1 s WAV files (16 kHz, mono, 16-bit) with Audacity.
➢ Pitch-based labelling was conducted manually by two professional annotators.
➢ MFCC matrices were produced with Praat and stored in .txt format.
➢ Images were generated with Librosa (Python) at a resolution of 1000 × 600.

### 4.2 Sound Segmentation and Pre-Processing

The Praat program, an extremely versatile program for acoustic analysis, assists the data collected to be separated into sections. Audacity assisted the conversion of recordings into one-second WAV files, whereas Praat was important in the acoustic analysis for the identification and classification of the SQEBSP dataset. The audio was preprocessed using Praat, including segmentation, labelling, and feature extraction.

**Fig. 2:**
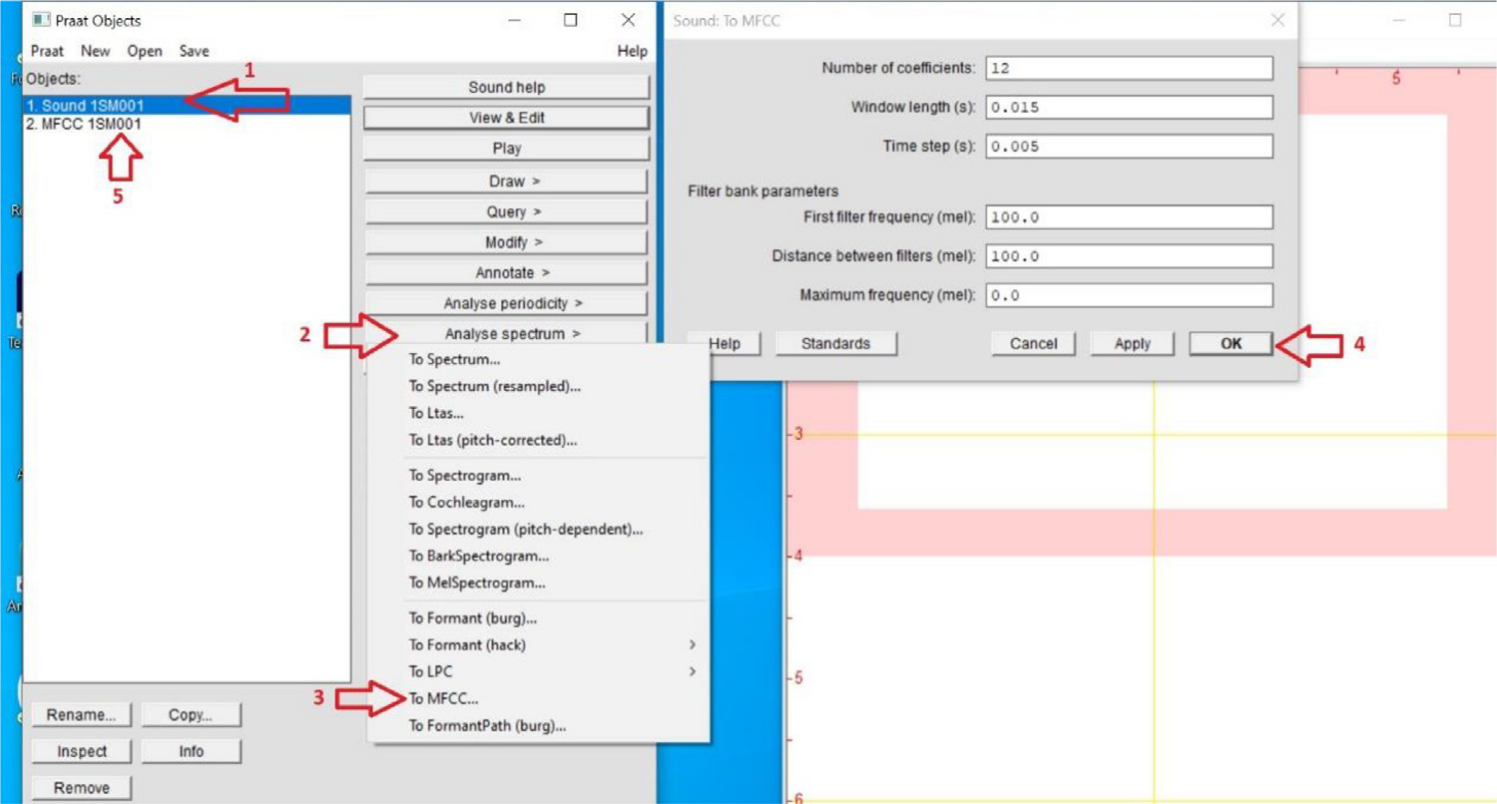
Example of spectral settings in Praat used to extract MFCCs.

Extraction of Mel-Frequency Cepstral Coefficients (MFCCs) was a fundamental part of the method. Fig. 2 shows the method of turning WAV files into MFCCs together with the particular Praat spectral analysis configuration used. This conversion turned every one-second audio segment into a matrix structure by letting the sound data be expressed in a format more fit for machine learning. Furthermore, the WAV files were transformed into image representations of the signal using the library in Python.

**Fig. 3:**
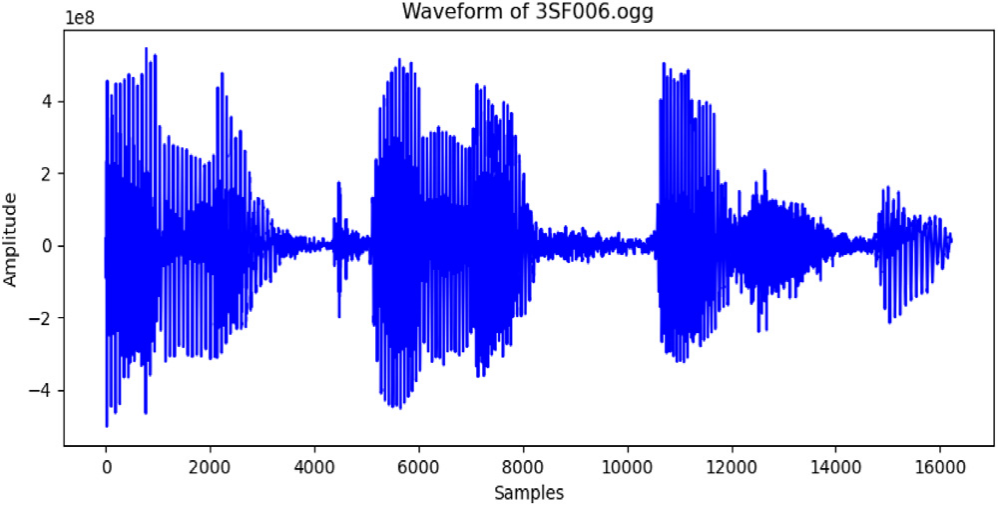
Image representation of wav file.

The WAV files were converted into graphical representations of the audio signals, which allowed their use as image-based datasets for sound recognition models, such as 2D Convolutional Neural Networks (CNNs). The conversion procedure employed the Librosa library in Python, generating images with a resolution of 1000 pixels in width and 600 pixels in height at 100 DPI, as illustrated in Fig. 3.

### 4.3 Labelling

Dataset labelling is essential for the creation and classification of new datasets. It includes providing important and precise labels to specific data within a dataset. The process involves the classification or identification of data based on particular criteria or characteristics. The proposed dataset labelling procedure included categorizing data into three phrase types: statements, questions, and exclamations. Each category contained 10 subdirectories corresponding to each phrase number, with each subdirectory containing over 420 data samples. The data was labelled to make it easier to separate male and female individuals. The researchers of this study performed the previously mentioned procedure for all four types of data: raw data (.ogg), WAV files, matrix files, and image files. The mechanism for data labelling is illustrated in Table 4.

**Table 4:** The procedure of labelling the dataset.

Phrase No.	Phrase type	Gender	File No.	Example	Extension
1: 10	S: Statement Q: Question E: Exclamation	M: for male F: for Female	1: 420+	1SM001 4QF231 6EM027	.ogg: Raw data .wav: Wave files .txt: Matrix files .png: Image file

## Limitations

Constructing a robust dataset for the classification of phrases and sentences faces restrictions related to availability of data, speaker variability, labelling challenges, limited scope, data privacy and ethical considerations, and scalability. These challenges can be classified into various categories. Limitations include restricted access to various data across phrase types, managing speaker-related variations, ensuring accurate annotations, reducing biases, considering the dataset's scope, and maintaining privacy and ethical standards. The quality and reliability of the sound collection for effective sentence identification research are guaranteed by addressing these limitations through precise collection, accurate annotations, ethical considerations, and scalability.

The other limitation noted during the preparation of the dataset was the hesitation of various people in recording their voices for dataset contribution. Most participants reported experiencing difficulty either because of privacy concerns or personal choice, which limited the ability to attain a wide-ranging and varied dataset. Also, it was difficult for some participants to provide 30 voice samples in a single session, and the authors had to compromise by allowing recording at different times or repeating it over a more manageable timeframe. These concerns affected the efficiency and timeline of the data collection, requiring flexibility and adjustment in the recording process.

## Ethics Statement

The ethical concerns of sound recognition datasets are informed consent of participants, confidentiality and data protection, awareness of culture, academic morality, organisational assessment, and sharing of data and access. These ethical guidelines protect participants' rights, provide authenticity to studies, and facilitate the sound and responsible development of phrase recognition sound datasets.

Our research team at the University of Halabja attempted to collect reliable data. We promise respect for strong ethical rules and regulations to ensure the validity, reliability, and accuracy of the data, in addition to the privacy of the participants. We agree to maintain transparency in our research and to provide reliable, applicable results to the scientific community.

To address ethical concerns, the research team created an effective informed agreement process with our Data Collection Contributors Information Form. The document clearly defined the research objectives, the proposed use of voice recordings, and the security measures implemented to ensure participants' privacy and confidentiality. By obtaining complete permission from each contributor and emphasising our adherence to strong ethical standards, our team at the University of Halabja guaranteed that data collection would be executed transparently and responsibly. This method protected the participants' rights and improved the validity, reliability, and integrity of our research results.

## CRediT authorship contribution statement

**Ayub Othman Abdulrahman:** Conceptualization, Supervision, Writing – original draft, Software. **Shanga Ismail Othman:** Validation, Data curation, Investigation, Writing – review & editing. **Gazo Badran Yasin:** Data curation, Methodology, Writing – review & editing. **Meer Salam Ali:** Visualization, Data curation, Writing – review & editing.

## Data Availability

Mendeley Data.A Dataset for Categorizing Phrases and Sentences as Statements, Questions, or Exclamations Depending on Sound Pitch (Original data)
